# Urine Bile Acids Relate to Glucose Control in Patients with Type 2 Diabetes Mellitus and a Body Mass Index Below 30 kg/m^2^

**DOI:** 10.1371/journal.pone.0093540

**Published:** 2014-04-15

**Authors:** David R. Taylor, Jamshid Alaghband-Zadeh, Gemma F. Cross, Sohail Omar, Carel W. le Roux, Royce P. Vincent

**Affiliations:** Department of Clinical Biochemistry, King's College Hospital, London, United Kingdom; Northeast Ohio Medical University, United States of America

## Abstract

Bile acids are important endocrine signalling molecules, modulating glucose homeostasis through activation of cell surface and nuclear receptors. Bile acid metabolism is altered in type 2 diabetes mellitus; however, whether this is of pathogenic consequence is not fully established. In this study urinary bile acid excretion in individuals with type 2 diabetes and matched healthy volunteers was assessed. Urinary bile acid excretion in type 2 diabetes patients was considered in the context of prevailing glycaemia and the patient body mass index. Urine bile acids were measured by liquid chromatography-tandem mass spectrometry, allowing individual quantification of 15 bile acid species. Urinary bile acid excretion in patients with type 2 diabetes who were normal weight (BMI 18.5–24.9 kg/m^2^) and overweight (BMI 25–29.9 kg/m^2^) were elevated compared to healthy normal weight volunteers, both p<0.0001. In obese (BMI≥30 kg/m^2^) type 2 diabetes patients, urinary bile acid excretion was significantly lower than in the normal and overweight type 2 diabetes groups (both p<0.01). Total bile acid excretion positively correlated with HbA1c in normal (rs = 0.85, *p* = <0.001) and overweight (rs = 0.61, *p* = 0.02) but not obese type 2 diabetes patients (rs = −0.08, *p* = 0.73). The glycaemia-associated increases in urine bile acid excretion in normal weight and overweight type 2 diabetes seen in this study may represent compensatory increases in bile acid signalling to maintain glucose homeostasis. As such alterations appear blunted by obesity; further investigation of weight-dependent effects of bile acid signalling on type 2 diabetes pathogenesis is warranted.

## Introduction

Bile acids are important endocrine molecules, initiating signalling by the nuclear farnesoid X receptor (FXR) and G-protein coupled receptor, TGR5 [1]. Bile acid signalling exerts diverse influence over glucose, lipid and energy homeostasis, functions additional to the classical role of bile acids in lipid solubilisation and absorption in the intestine [2].

In the L-cells of the intestinal epithelium, TGR5 activation by bile acids stimulates the release of gut hormones such as glucagon-like peptide-1 (GLP-1) [3], resulting in improved glucose homeostasis [4]. In plasma, bile acid concentrations rise rapidly in response to glucose ingestion, which likely underpins many of the mechanisms by which bile acids play a role in regulation of the body's response to food intake [5]. In pancreatic β-cells TGR5 and FXR activation enhances insulin secretion [6], [7],whilst in the liver, FXR activation may inhibit gluconeogenesis, whilst increasing glycogen synthesis and improving hepatic insulin sensitivity, although a role for FXR in gluconeogenesis remains controversial [1]. Glucose itself stimulates expression of cholesterol 7α-hydroxylase, which catalyses the rate-limiting step in bile acid production [8], [9].

Many diabetes-associated changes in bile acid metabolism have been reported; these include altered synthetic rate (both increased and decreased synthesis reported), pool size and composition of bile acid species [10]–[14]. Lowering of the bile acid pool size using a synthetic FXR agonist reduces energy expenditure and induces obesity and diabetes in mice [15]. Moreover, bile acid sequestrant therapy improves glycaemic control in type 2 diabetes, in addition to its lipid-lowering action [16], [17].

Obesity increases the risk of type 2 diabetes mellitus, and the prevalence of both continues to rise annually [18]. Weight loss, by changes in lifestyle or metabolic surgery, improves glycaemia in type 2 diabetes [19]–[21].The beneficial impact of Roux-en-Y gastric bypass surgery on diabetes control is associated with enhanced β-cell function and improved insulin sensitivity which has been attributed, in part, to an exaggerated post-prandial GLP-1 response [22]–[24]. Fasting plasma bile acids are lower in overweight and severely obese individuals compared to those post-RYGB [25]. Further, obesity blunts post-prandial plasma bile acid response relative to responses in normal weight individuals [26], although obese patients with type 2 diabetes mellitus exhibit greater post-prandial response of plasma bile acid compared to normoglycaemic obese patients [27].

Despite the large body of literature supporting a critical role for bile acids in glucose homeostasis in health and disease, the precise relationship between bile acids and prevailing glycaemia in type 2 diabetes remains ill defined, as does whether this relationship is affected by body weight. Therefore, in this study we investigated the relationship of urinary bile acid excretion with glycated haemoglobin (HbA1c), in a well characterised cohort of people with type 2 diabetes mellitus. Bile acids escaping first pass hepatic clearance reach the circulation and a proportion are subject to glomerular filtration [28]. Whilst up to 97% of the bile acids are recaptured in the proximal renal tubule via the membrane transporter proteins ASBT and OSTα, a small amount ends up in urine. Importantly, urinary bile acid concentration, when expressed as a creatinine ratio, correlates extremely well with fasting plasma values [29]. Therefore, urine provides an excellent, non-invasive matrix in which to investigate glycaemia-associated changes in bile acid concentrations. We hypothesised that in patients with type 2 diabetes there is a compensatory increase of bile acids, possibly mediated by an increase in plasma glucose. Furthermore, we hypothesised that this compensatory increase may be influenced by weight gain.

## Methods and Materials

### Ethics statement

The study was approved by the King's College Hospital NHS Foundation Trust Research Ethics Committee (reference number 05/Q0703/4). Written informed consent was obtained from all participants in the study.

### Samples

Fifty urine samples from patients with type 2 diabetes and 15 from non-diabetic controls were studied. Urine bile acid measurements were performed using early morning first pass urine samples taken for clinical purposes. Sample aliquots were frozen on the same day and stored at −20°C prior to analysis. Electronic patient records were accessed to confirm the people with diabetes were treated by only lifestyle change and/or metformin, had normal albumin:creatinine ratios (men <2.5 mg/mmol, women <3.5 mg/mmol) and had date-matched HbA1c and body mass index (BMI) measurements. All study participants had normal renal function (eGFR>90 mL/min/1.73 m^2^), normal thyroid function (thyroid stimulating hormone 0.3–5.5 mIU/L) and normal liver function (albumin 35–50 g/L, aspartate aminotransferase <50 IU/L, alkaline phosphatase <130 IU/L, bilirubin <20 µmol/L). Patients using insulin, GLP-1 receptor analogue or dipeptidyl peptidase 4 (DDP4) inhibitor therapies were excluded. The samples were grouped by BMI of the donor, according to World Health Organisation criteria (BMI<24.99 kg/m^2^ =  normal; 25–29.99 kg/m^2^ =  overweight, obese  = ≥30 kg/m^2^) [30]. Statin use across type 2 diabetes subgroups was uniform. Control samples were obtained from 12 non-diabetic volunteers, recruited from the laboratory.

### Assay methods

HbA1c was measured by boronate affinity chromatography using a Primus Ultra2 HPLC system (Primus Diagnostics, Kansas City, USA). Urine creatinine measurement was performed using a kinetic Jaffé reaction on an Advia 2400 analyser (Siemens Healthcare Diagnostics, Frimley, UK).

### Bile acid conjugate hydrolysis

Urinary bile acid profiles exhibit great complexity [31], [32]. Glucuronidated and sulphated species are found at higher concentrations than in plasma, and would not be detected by the mass spectrometry method used, which only measures glycine-, taurine- and unconjugated cholic, chenodeoxycholic, deoxycholic, ursodeoxycholic and lithocholic acid species. Thus, enzymatic digestion using *Helix pomatia* extract (which contains high sulphatase and glucuronidase activities) was utilised to remove glucuronide and sulphate conjugations using an existing protocol [33]. Briefly, internal standard was added to 10 mL urine and the urine then extracted on Sep-Pak columns, eluted in methanol and dried under nitrogen. Urine residues were then dissolved in 5 mL 0.5 M sodium acetate containing 10 mg/mL of *H. pomatia* powder and 20 mg/mL of sodium ascorbate (to inhibit cholesterol oxidase activity) for 72 h at 37°C. Freed bile acids were then re-extracted on Sep-Pak columns, eluted and dried. Dried solvolysis and enzyme-treated samples were re-suspended in 250 µL of a 50∶50 (v/v) mix of mobile phases A and B. 10 µL of this solution was injected onto the column (equal to 0.4 mL of the original urine sample).

### High performance liquid chromatography tandem mass spectrometry method

Bile acid measurement was carried out using an existing high performance liquid chromatography tandem mass spectrometry (LC-MS/MS) method [26], [34]. The method uses an Acentis fused-core C18 analytical column (150×4.6 mm, particle size 2.7 µm, Sigma-Aldrich) on a Jasco LC2000 series HPLC system attached to an API 3200 triple quadrupole mass spectrometer (Applied Biosystems, Cheshire, UK). Mobile phases comprised (A) methanol or (B) deionised H_2_O, each containing 5 mM ammonium acetate (w/v) and 0.012% formic acid (v/v). Negative ion mass spectra of the fractionated bile acids were recorded in multiple reaction-monitoring mode. Data were captured using Analyst Software version 1.4.2 (Applied Biosystems) and quantitation carried out by peak area analysis corrected to internal standards (a 2,2,4,4,d4-deoxycholic acid for each of the bile acid chemistries; unconjugated, glycine- and taurine-conjugated). For quantitation, a bile acid calibration curve was prepared using bile acid standards dissolved in charcoal-stripped urine in the concentration range 500–20,000 nmol/L. Urine bile acid concentrations in the study group were normalised to urinary creatinine concentration. Lower limit of quantitation for each bile acid was 0.1 µmol/L, intraassay coefficients of variation for each bile acid were in the range 2.8–12.1% whilst interassay precisions were between 1.6–13.6% [26].

### Presentation of bile acid profile: the bilogram

Bile acid data are presented according to biological groupings in a bilogram. The primary bile acids cholic and chenodeoxycholic acids are synthesised in the liver under the control of 7α-hydroxylase. These can be conjugated with either glycine or taurine, giving rise to four primary conjugated species. Bacterial action in the intestine leads to the formation of the secondary bile acids deoxycholic acid, lithocholic acid and ursodeoxycholic acid. Each can be conjugated with glycine or taurine giving rise to six secondary-conjugated bile acids. The bilogram thus shows the primary, primary conjugated, secondary and secondary conjugated bile acids. Total bile acid, total unconjugated, total glycine-conjugated and total taurine-conjugated values are also given in the bilogram.

### Statistical analysis

Statistical analysis was performed using Analyse-It® (version 2.21, Leeds, U.K.). Data were tested for normality using a Shapiro-Wilk test and were not normally distributed. Comparison across multiple groups was performed using Kruskal-Wallis ANOVA. Pair-wise comparisons were performed using Mann-Whitney U tests with post-hoc Bonferroni correction. Spearman correlation plots were produced for urinary bile acid excretion with various variables. Data is reported as median and interquartile ranges (IQR).Values of P<0.05 were taken as statistically significant.

## Results

The demographics of the sample donors are shown in table 1. The 15 control volunteers were aged 50 (29.3–61) [median (IQR)] years and had a BMI of 23 (20–24) kg/m^2^. The 50 type 2 diabetes mellitus patients were aged 62 (47–74) years and had a BMI of 30 (27–32) kg/m^2^ and HbA1c of 8.1 (6.8–9.1)% [65.5 (51.0–76.2) mmol/mol]. BMI, age, HbA1c and urinary creatinine differences did not reach statistical significance between the groups (Table 1). The bilogram shown in Table 2 details urinary bile acid excretion in both groups. The total bile acid excretion was significantly higher in the type 2 diabetes group at 877.4 (406.7–1454.5) nmol/mmol creatinine than in the control group at 124.6 (53.1–172.5) nmol/mmol creatinine (p = <0.0001) (Table 2). Statistically significant differences in urinary bile acid excretion between control and type 2 diabetes mellitus samples were observed in 13 of the 15 individual bile acid species measured (Table 2). In both groups, the glycine-conjugated bile acids were the most abundant species, with the secondary bile acid glycodeoxycholic acid being excreted in the highest amounts (Table 2).

**Table 1:** Demographics of the study cohort.

	Healthy volunteers	All T2DM data	Normal BMI T2DM	Overweight T2DM	Obese T2DM	P value
Number	15	50	14	15	21	-
Male/female	8/7	30/20	10/4	8/7	12/9	NS†
Age (years)	50.0 (29.3–61.0)	61.0 (45.9–75.0)	53.0 (42.8–66.4)	65.0 (48.8–75.0)	61.0 (47.7–77.3)	NS
BMI (kg/m^2^)	23 (20–24)	30 (27–32)	23 (20–25)	28 (27–30)	34 (32–40)	-
HbA1c % (mmol/mol Hb)	Not measured	8.1 (6.8–9.1) [*65.5 (51.0–76.2)*]	6.6 (5.9–8.7) [*48.5 (40.8–71.4)*]	8.2 (7.4–9.5) [*66.0 (57.2–80.2)*]	8.2 (7.3–9.0) [*66.0 (56.7–75.3)*]	NS
Urine creatinine (mmol/L)	8.7 (6.4–16.7	7.8 (5.5–12.6)	10.7 (5.2–17.0)	7.9 (6.2–12.5)	7.5 (5.4–9.1)	NS

Data presented as median and interquartile range. Abbreviations: T2DM, type 2 diabetes mellitus; BMI, body mass index; HbA1c, glycated haemoglobin; NS, not significant. P values were obtained by Kruskal-Wallis ANOVA and Chi-square test (†). P<0.05 was taken as statistically significant.

**Table 2:** Median urinary bile acid excretion in healthy volunteers and type 2 diabetes mellitus.

Bile acid	Healthy volunteers	All T2DM data	*P* value healthy vs T2DM
Total bile acids	124.6 (53.1–172.5)	877.4 (406.7–1454.5)	<0.0001
Total unconjugated	14.7 (6.9–24.1)	128.7 (84.1–235.3)	<0.0001
Total glycine-conjugated	88.3 (43.6–126.1)	586.5 (281.7–1034.9)	<0.0001
Total taurine-conjugated	19.8 (4.2–27.1)	60.2 (17.8–130.3)	0.0008
***Primary***			
Cholic acid	2.4 (1.6–6.9)	34.0 (12.9–71.4)	<0.0001
Chenodeoxycholic acid	1.0 (0.4–3.1)	10.5 (5.6–29.0)	<0.0001
***Primary conjugated***			
Glycocholic acid	16.1 (12.6–27.4)	44.4 (17.2–128.5)	0.003
Taurocholic acid	2.2 (1.2–2.9)	6.0 (2.1–13.4)	0.01
Glycochenodeoxycholic acid	11.5 (5.3–21.7)	100.4 (42.3–148.1)	<0.0001
Taurochenodeoxycholic acid	3.7 (1.6–7.3)	7.1 (3.3–17.3)	NS
***Secondary***			
Deoxycholic acid	6.0 (3.8–8.4)	42.0 (15.3–91.3)	<0.0001
Ursodeoxycholic acid	1.8 (1.1–3.5)	10.1 (4.6–26.1)	<0.0001
Lithocholic acid	0.6 (0.2–1.8)	8.6 (4.5–15.8)	<0.0001
***Secondary conjugated***			
Glycodeoxycholic acid	25.1 (15.6–39.2)	187.6 (82.0–433.1)	<0.0001
Taurodeoxycholic acid	1.9 (0.8–3.6)	10.5 (2.4–22.9)	0.0006
Glycoursodeoxycholic acid	5.3 (2.8–13.4)	58.9 (22.5–94.7)	<0.0001
Tauroursodeoxycholic acid	0.9 (0.6–1.3)	2.1 (0.75–9.0)	NS
Glycolithocholic acid	10.4 (7.0–24.7)	118.1 (27.1–256.0)	<0.0001
Taurolithocholic acid	5.3 (1.0–8.5)	18.0 (3.4–45.8)	0.006

Urinary bile acid excretion expressed as nmol bile acid/mmol creatinine. Data presented as median and inter-quartile range. Mann-Whitney tests were used to compare differences in urinary bile acid excretion between the two groups. NS, not significant. P<0.05 was taken as statistically significant.

When the type 2 diabetes mellitus group was subdivided according to BMI, Kruskal-Wallis ANOVA revealed there to be significant differences in urinary bile acid excretion between the healthy volunteers and normal weight, overweight and obese type 2 diabetes groups. Comparison of the healthy controls to either the normal weight or overweight type 2 diabetes mellitus groups demonstrated significant increases in urinary bile acid excretion in type 2 diabetes for all bile acid species, with the exception of taurochenodeoxycholic acid (controls vs. normal weight or overweight type 2 diabetes) and tauroursodeoxycholic acid (controls vs. overweight type 2 diabetes) (Table 3). In contrast, when urinary bile acid excretion in healthy volunteers and obese type 2 diabetes was compared, significant increase in excretion were seen in only seven out of 15 bile acid species studied (cholic acid, chenodeoxycholic acid and its glycine conjugate, deoxycholic acid and its glycine conjugate, ursodeoxycholic acid and its glycine conjugate and lithocholic acid and its glycine conjugate). Next, urinary bile acid excretion in normal weight type 2 diabetes was compared to that in both the overweight and obese type 2 diabetes groups. There was no difference in excretion between the normal weight and overweight type 2 diabetes groups (Table 3). However, urinary bile acid excretion in the obese type 2 diabetes group was significantly lower for the bile acids glycochenodeoxycholic acid, deoxycholic acid (and its glycine conjugate) and glycine and taurine-conjugated ursodeoxycholic acid, relative to normal weight type 2 diabetes (Table 3). Similar results were seen when the overweight and obese type 2 diabetes mellitus groups were compared, with the exception that cholic acid and its taurine conjugate, as well as glycolithocholic acid, were also significantly lower in the obese (Table 3). Relative proportions of primary and secondary bile acids were not different between control and type 2 diabetes samples (irrespective of patient BMI) (Table 3).

**Table 3:** Comparison of median urinary bile acid excretion between healthy volunteer and type 2 diabetes mellitus groups classified according to BMI.

					*P* value					
Bile acid	Healthy volunteers	Normal weight T2DM	Overweight T2DM	Obese T2DM	Volunteer vs normal weight T2DM	Volunteer vs overweight T2DM	Volunteer vs obese T2DM	Normal weight T2DM vs overweight T2DM	Normal weight T2DM vs obese T2DM	Overweight T2DM vs obeseT2DM
Total bile acids	124.6 (53.1–172.5)	1298.6 (969.2–1708.9)	1163.2 (503.8–2713.6)	385.9 (190.0–719.3)	<0.0001	<0.0001	<0.001	NS	<0.01	<0.01
Total unconjugated	14.7 (6.9–24.1)	123.1 (130.7 288.3)	158.7 (102.0–313.7)	84.6 (47.3–125.2)	<0.0001	<0.0001	<0.001	NS	<0.01	0.03
Total glycine-conjugated	88.3 (43.6–126.1)	1027.0 (731.4–1268.3)	788.7 (357.6–1238.8)	258.9 (113.7–505.6)	<0.0001	<0.0001	<0.01	NS	<0.01	<0.01
Total taurine-conjugated	19.8 (4.2–27.1)	184.7 (51.7–150.4)	62.7 (40.4–183.2)	18.2 (9.3–85.2)	<0.0001	<0.001	NS	NS	0.02	NS
***Primary***								NS		
Cholic acid	2.4 (1.6–6.9)	35.7 (18.3–65.0)	58.7 (35.8–126.6)	12.9 (6.9–47.8)	<0.0001	<0.0001	0.002	NS	NS	<0.01
Chenodeoxycholic acid	1.0 (0.4–3.1)	10.5 (7.2–29.0)	16.2 (7.4–30.8)	7.2 (2.0–25.1)	<0.0001	<0.0001	0.002	NS	NS	NS
***Primary conjugated***								NS		
Glycocholic acid	16.1 (12.6–27.4)	72.9 (24.6–173.8)	72.0 (28.8–166.3)	34.6 (12.7–54.4)	0.007	0.002	NS	NS	NS	NS
Taurocholic acid	2.2 (1.2–2.9)	12.4 (3.5–19.0)	10.3 (4.6–25.5)	5.6 (1.3–7.6)	0.001	<0.01	NS	NS	NS	0.04
Glycochenodeoxycholic acid	11.5 (5.3–21.7)	135.8 (82.1–148.1)	140.4 (65.2–246.7)	56.0 (19.6–103.3)	<0.0001	<0.0001	0.002	NS	0.01	0.03
Taurochenodeoxycholic acid	3.7 (1.6–7.3)	10.0 (4.2–37.5)	8.5 (4.6–33.6)	4.0 (1.8–8.2)	NS	NS	NS	NS	NS	NS
***Secondary***										
Deoxycholic acid	6.0 (3.8–8.4)	87.0 (51.5–107.6)	51.1 (34.5–122.5)	16.3 (12.6–37.5)	0.0003	<0.0001	<0.01	NS	<0.01	<0.01
Ursodeoxycholic acid	1.8 (1.1–3.5)	20.2 (10.0–31.9)	9.3 (7.1–19.8)	6.5 (4.1–32.6)	<0.0001	<0.0001	0.002	NS	NS	NS
Lithocholic acid	0.6 (0.2–1.8)	9.7 (5.8–15.6)	11.1 (6.7–18.6)	5.0 (0.8–15.8)	<0.0001	<0.0001	0.03	NS	NS	NS
***Secondary conjugated***										
Glycodeoxycholic acid	25.1 (15.6–39.2)	455.1 (236.2–641.6)	243.7 (112.9–480.0)	60.9 (39.5–187.0)	<0.0001	<0.0001	0.002	NS	<0.01	NS
Taurodeoxycholic acid	1.9 (0.8–3.6)	15.1 (6.1–27.2)	14.1 (6.7–19.7)	3.7 (0.8–19.5)	<0.0001	0.0004	NS	NS	NS	NS
Glycoursodeoxycholic acid	5.3 (2.8–13.4)	80.7 (43.6–123.7)	67.3 (29.1–131.6)	22.2 (11.8–63.0)	<0.0001	<0.0001	0.005	NS	<0.01	0.02
Tauroursodeoxycholic acid	0.9 (0.6–1.3)	6.5 (1.6–16.8)	4.0 (0.5–8.1)	1.1 (0.3–2.5)	0.002	NS	NS	NS	NS	NS
Glycolithocholic acid	10.4 (7.0–24.7)	208.6 (58.7–300.1)	122.3 (108.5–335.0)	34.6 (22.8–144.8)	0.001	<0.0001	0.02	NS	NS	0.03
Taurolithocholic acid	5.3 (1.0–8.5)	26.5 (8.5–51.4)	21.0 (9.6–61.0)	4.5 (1.5–40.1)	0.003	0.002	NS	NS	NS	NS

Urinary bile acid excretion expressed as nmol bile acid/mmol creatinine. Mann-Whitney test with Bonferroni correction (following prior Kruskal-Wallis ANOVA) was used to compare differences in excretion between 1) healthy volunteers and T2DM subgroups and 2) in the BMI 25–30 and BMI>30 groups relative to normal weight T2DM (BMI<25). Individual P values are indicated. P<0.05 was taken as statistically significant. NS  =  not significant.

**Figure 1:**
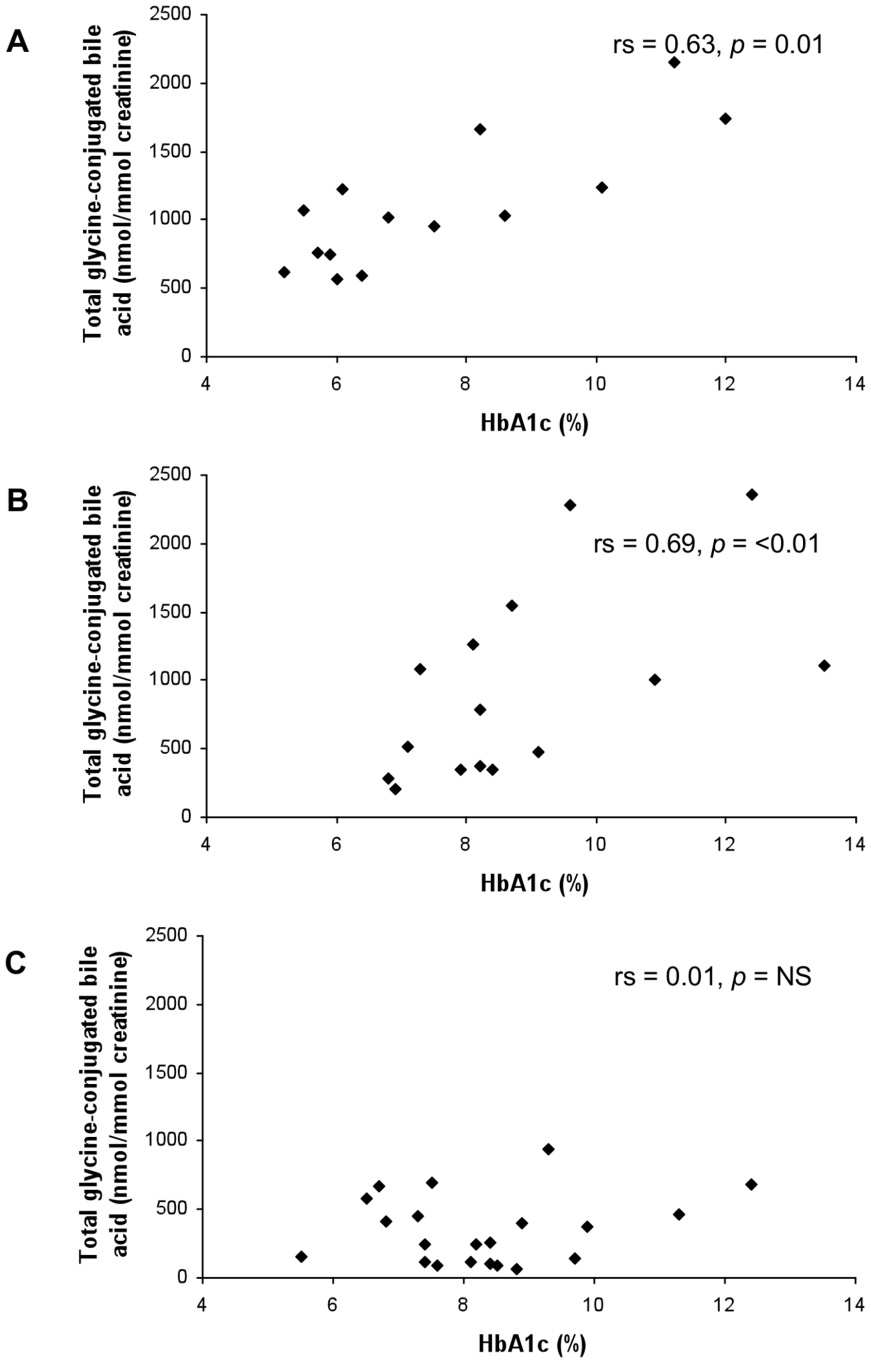
HbA1c plotted against total glycine-conjugated bile acid urinary excretion for normal weight (A), overweight (B) and obese (C) type 2 diabetes mellitus groups.

To assess whether the increased urinary bile acid excretion seen in type 2 diabetes mellitus was related to glycaemia, Spearman correlation plots were constructed for HbA1c with urine bile acid data (Table 4). When all type 2 diabetes data were considered, no correlation with HbA1c was observed (Table 4). However, in normal weight type 2 diabetes, total, total unconjugated and total glycine-conjugated urinary bile acid excretion were positively correlated with HbA1c (Table 4, Figure 1, representative graphs for total glycine conjugated bile acid excretion). In contrast, taurine-conjugated urinary bile acid excretion was not associated with glycaemic control (Table 4). The secondary bile acid deoxycholic acid and its glycine conjugate, as well as lithocholic acid demonstrated the most statistically significant correlation with glycaemia.

**Table 4:** Correlation of urinary bile acid excretion with glycaemic control in type 2 diabetes mellitus.

	All T2DM data		Normal weight T2DM		Overweight T2DM		Obese T2DM	
	rs	*P*	rs	*P*	rs	*P*	rs	*P*
Total bile acids	0.19	0.18	**0.85**	**<0.001**	**0.61**	**0.02**	−0.08	NS
Total unconjugated	0.24	0.09	**0.83**	**<0.001**	**0.62**	**0.01**	−0.05	NS
Total glycine-conjugated	0.18	0.21	**0.69**	**<0.01**	**0.63**	**0.01**	0.01	NS
Total taurine conjugated	0.03	0.82	−0.12	NS	0.44	NS	−0.14	NS
***Primary***								
Cholic acid	0.08	0.57	0.13	NS	**0.56**	**0.03**	−0.16	NS
Chenodeoxycholic acid	0.11	0.43	0.27	NS	**0.59**	**0.02**	−0.25	NS
***Primary conjugated***								
Glycocholic acid	0.09	0.53	−0.14	NS	0.47	NS	0.06	NS
Taurocholic acid	0.08	0.60	−0.24	NS	**0.63**	**0.01**	−0.07	NS
Glycochenodeoxycholic acid	0.13	0.37	0.05	NS	0.50	NS	0.17	NS
Taurochenodeoxycholic acid	0.03	0.83	−0.06	NS	**0.57**	**0.03**	−0.26	NS
***Secondary***								
Deoxycholic acid	0.14	0.33	**0.82**	**<0.001**	0.41	NS	−0.38	NS
Ursodeoxycholic acid	0.05	0.71	0.03	NS	0.40	NS	−0.01	NS
Lithocholic acid	0.25	0.08	**0.56**	**0.04**	**0.75**	**<0.01**	−0.17	NS
***Secondary conjugated***								
Glycodeoxycholic acid	0.17	0.2433	**0.81**	**<0.001**	**0.70**	**<0.01**	−0.18	NS
Taurodeoxycholic acid	0.04	0.7839	0.11	NS	0.24	NS	−0.08	NS
Glycoursodeoxycholic acid	0.13	0.38	0.30	NS	0.28	NS	0.15	NS
Tauroursodeoxycholic acid	−0.09	0.55	−0.44	NS	0.20	NS	0.09	NS
Glycocholic acid	0.09	0.53	−0.14	NS	0.47	NS	0.06	NS
Taurolithocholic acid	0.07	0.61	0.28	NS	0.35	NS	−0.31	NS

Spearman correlation plots were constructed for urinary bile acid excretion and HbA1c, the preferred marker of glycaemic control. Correlation (rs) and P values are indicated. P<0.05 was taken as statistically significant. NS  =  not significant.

**Figure 2:**
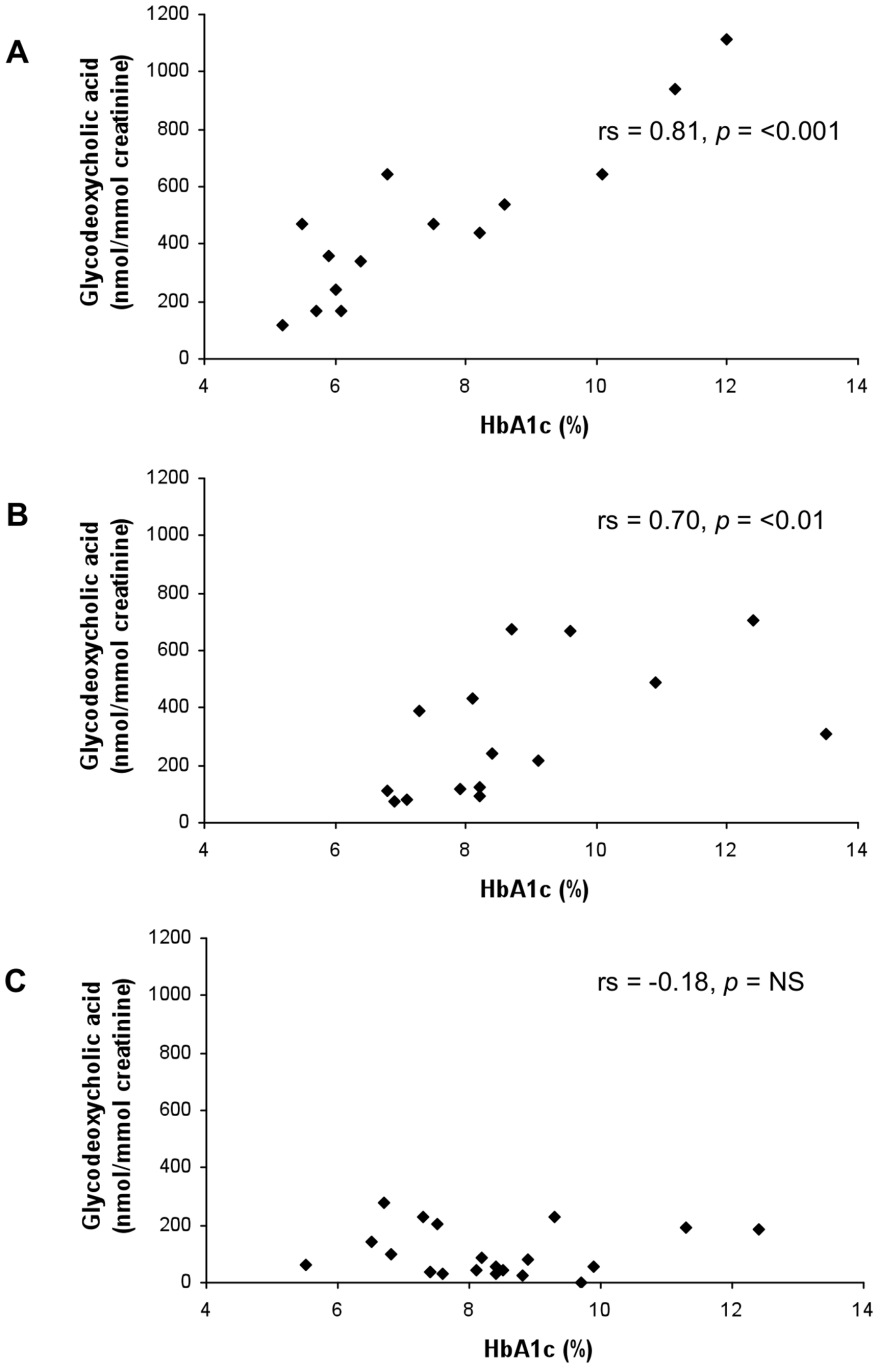
HbA1c plotted against glycodeoxycholic acid urinary excretion for (A) normal weight, (B) overweight and (C) obese type 2 diabetes mellitus groups.

In the overweight type 2 diabetes group, total, total unconjugated and total glycine-conjugated urinary bile acid excretion showed positive correlation with HbA1c; however, the correlation was not as strong as for normal weight type 2 diabetes mellitus (Table 4). In this group, significant correlations between the primary bile acids cholic acid and chenodeoxycholic acid, and the secondary bile acids glycodeoxycholic acid, lithocholic acid and glycolithocholic acid with glycaemic control were seen (Table 4, Figure 2).

**Figure 3:**
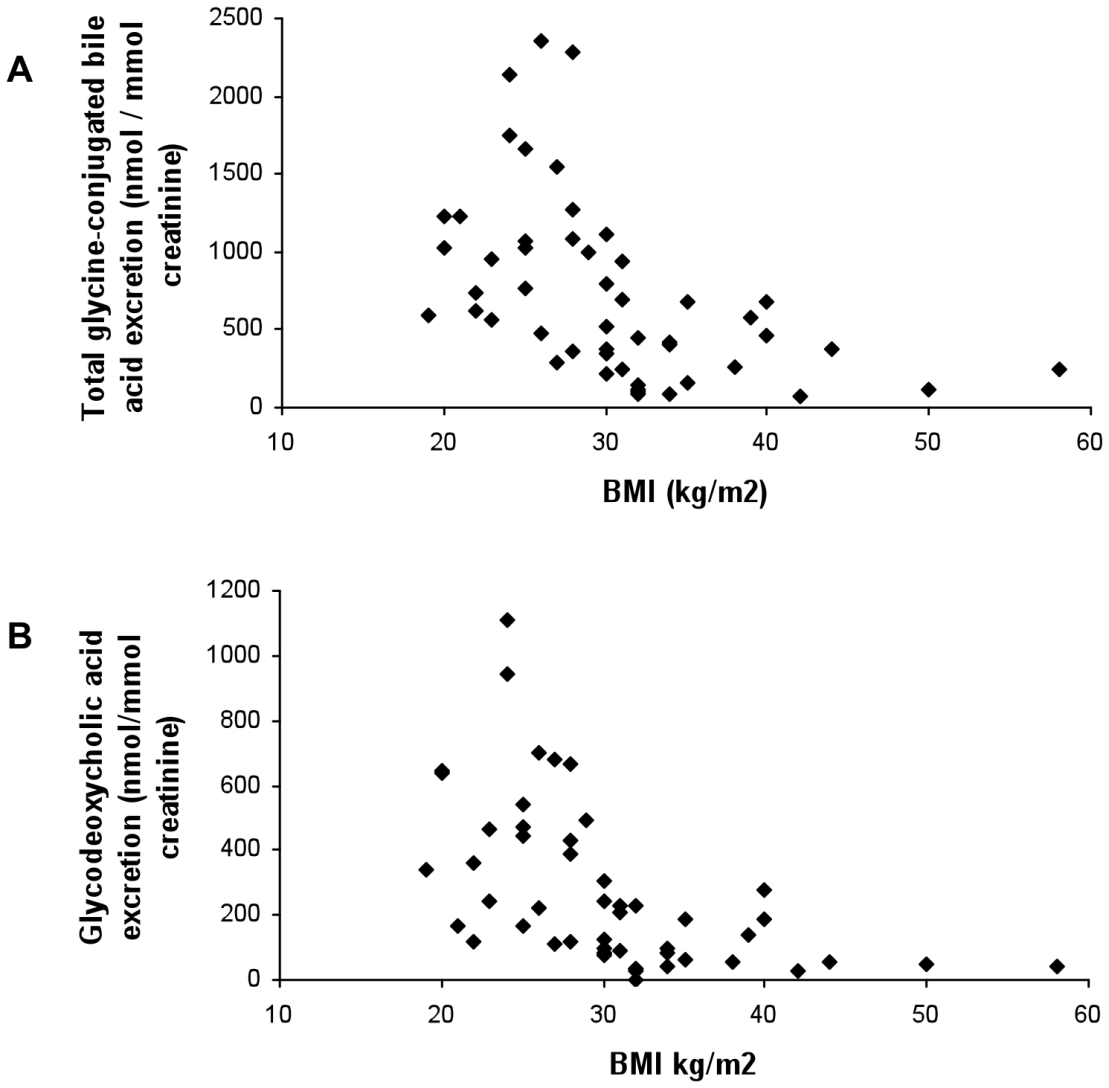
Graph plotting BMI of patient against total glycine-conjugated (A) and glycodeoxycholic acid (B) urinary bile acid excretion.

Strikingly, correlation of urinary bile acid excretion (both total and individual species) with glycaemic control was lost altogether in patients classified as obese (Table 4, Figures 1 and 2). As a demonstration of the differences in correlation of glycaemic control with urinary bile acid excretion between the different BMI subgroups, graphs for total glycine-conjugated bile acid (Figure 1) and glycodeoxycholic acid (Figure 2) excretion are shown. When BMI was plotted against urinary bile acid excretion, there was a marked negative correlation of urinary bile acid excretion with BMI≥30 kg/m^2^ (Figure 3). Indeed, urinary bile acid excretion was almost undetectable in morbidly obese individuals (Figure 3).

## Discussion

In this study we demonstrate that urinary bile acid excretion is increased in type 2 diabetes mellitus compared to healthy controls, and that this increase is most evident in normal weight and overweight type 2 diabetic patients. Moreover, despite the study groups being relatively small, we clearly show that urinary bile acids are related to glycaemia, reflected by HbA1c, in normal weight and overweight, but not obese type 2 diabetic patients. Finally, we show that in type 2 diabetes, urinary bile acid excretion is suppressed at higher BMI.

Gross rearrangements of bile acid metabolism in type 2 diabetes are well established, with altered synthetic rate [10], [11], pool size [12], [13] and individual species composition [10], [14]. Given the emerging evidence that bile acids are important regulators of glucose homeostasis [2], such alterations may reflect altered bile acid signalling in type 2 diabetes mellitus pathogenesis in humans. In support, a recent study demonstrated insulin resistance in healthy subjects is associated with increased 12-hydroxylated bile acid species including cholic and deoxycholic acid [35], Nonetheless in type 2 diabetes the relationship between bile acids and prevailing glycaemic control remains poorly defined, as does its dependence of BMI. In the present study, urine samples were used to study whether bile acid turnover is related to HbA1c as a marker of glycaemic control. As urinary bile acid excretion expressed as a creatinine ratio correlates tightly with fasting plasma bile acid concentration [29], its measurement on early morning first pass urine samples represents an excellent non-invasive test for this purpose. Moreover, urinary bile acid excretion has been shown to increase in both human and animal studies of diabetes mellitus, providing support for the validity of the findings of glycaemia-associated changes in urinary bile acid excretion in type 2 diabetes in this study [14], [36]. Plasma bile acid measurement, on the other hand, was not performed owing to large intra-individual biological variation over a 24 hour period (23% to 91%) [37]. Such variation largely reflects increased post-prandial bile acid concentrations [5].

The increased bile acid excretion in people with type 2 diabetes mellitus is compatible either with an impact of diabetes on bile acid turnover, or with the increase in bile acid production being a compensatory response to the diabetic milieu to maintain glucose homeostasis. Post-prandial release of the incretin GLP-1 is impaired in type 2 diabetes mellitus [38], and HbA1c inversely correlates with maximal GLP-1 release [39]; suggesting that poorer glycaemic control is associated with a greater defect in incretin function. In normal physiology, bile acid activation of TGR5 stimulates GLP-1 release [3], [4], and plasma glycochenodeoxycholic acid and glycodeoxycholic acid concentrations correlate with GLP-1 after a mixed meal test [40]. Moreover, concomitant intra-jejunal infusion of both taurocholic acid and glucose was shown to increase both GLP-1 and C-peptide:glucose ratio relative to glucose infusion alone in healthy volunteers [41]. Therefore, the increased bile acid turnover in type 2 diabetes seen in this study may suggest the existence of a positive feedback mechanism to increase GLP-1 response. Glucose stimulates bile acid production by increasing *CYP7A1* expression [8], [9] which may provide a mechanism for the increased bile acid excretion in poorly controlled type 2 diabetes mellitus, and the correlation we have found between bile acid excretion and HbA1c. Increasing GLP-1 release by bile acid -mediated mechanisms would be expected to be beneficial in type 2 diabetes mellitus. In support, bile acid sequestrants structurally mimic bile acids and improve glycaemic control by a TGR5-dependent mechanism [42], [43].

In our study, the relationship between bile acid excretion and glycaemia appears lost in obese type 2 diabetic patients, as urinary bile acid excretion is suppressed in this group. Obesity is a state of low grade chronic inflammation, inducing insulin resistance in target tissues [44], [45]. Cytokines are known to reduce bile acid synthesis by direct inhibition of *CYP7A1* expression [46]. Thus, low urinary bile acid excretion in obese type 2 diabetic patients may reflect cytokine suppression, which in turn may have damaging effects on glucose homeostasis. In support of this, post-prandial plasma bile acid response is blunted in the obese [26], and several bile acid metabolites are associated with insulin sensitivity [47]. Chemically reducing bile acid pool size in animal models induces both diabetes mellitus and obesity [15], supporting the hypothesis that the suppressed urinary bile acid output seen in the obese in this study is of pathological significance.

One of the limitations of the study is the small cohort. The small numbers were the consequence of the strict selection criteria applied to try to avoid any influence of confounding factors on bile acid metabolism. Despite this, study numbers were sufficient to demonstrate the profound glycaemia-related changes in bile acid metabolism in normal weight and overweight type 2 diabetes.

The data in this study argue that at least for the obese type 2 diabetes patients, restoration of bile acid metabolism may be beneficial. Weight loss is associated with improvements in bile acid metabolism [25], [48]. Bariatric surgery provides the most significant and durable weight loss, but remission of type 2 diabetes mellitus precedes significant weight loss. This may be associated with enhanced β-cell function, hepatic insulin sensitivity, and nutrient rerouting, which many studies have causally related to the improved post-prandial GLP-1 responses [22]–[24]. Fasting bile acids are dramatically increased post-RYGB relative to obese controls [25], [48], and may thus contribute to type 2 diabetes remission perhaps via GLP-1. Weight loss by dietary means alone has also been shown to improve post-prandial GLP-1 response in the obese although bile acids were not measured [49].

In summary, the data in this study show that bile acids are increased in type 2 diabetes mellitus as a function of glycaemia in individuals with a BMI below 30 kg/m^2^. This is consistent with glucose stimulating bile acid production to maintain glucose homeostasis, possibly by increased GLP-1 response and altering FXR mediated signalling. Understanding how these compensatory mechanisms are lost in obese individuals with type 2 diabetes may provide an avenue for the development of new therapeutic strategies.
